# Precise mapping of the transcription start sites of human microRNAs using *DROSHA* knockout cells

**DOI:** 10.1186/s12864-016-3252-7

**Published:** 2016-11-11

**Authors:** Geon Jeong, Yeong-Hwan Lim, Young-Kook Kim

**Affiliations:** 1 Department of Biochemistry, Chonnam National University Medical School, Gwangju, Korea; 2 Center for Creative Biomedical Scientists, Chonnam National University Medical School, Gwangju, Korea

**Keywords:** MicroRNA, Promoter, Transcription start site, DROSHA, Knockout

## Abstract

**Background:**

The expression of microRNAs (miRNAs) is primarily regulated during their transcription. However, the transcriptional regulation of miRNA genes has not been studied extensively owing to the lack of sufficient information about the promoters and transcription start sites of most miRNAs.

**Results:**

In this study, we identified the transcription start sites of human primary miRNAs (pri-miRNAs) using *DROSHA* knockout cells. *DROSHA* knockout resulted in increased accumulation of pri-miRNAs and facilitated the precise mapping of their 5′ end nucleotides using the rapid amplification of cDNA ends (RACE) technique. By analyzing the promoter region encompassing the transcription start sites of miRNAs, we found that the unrelated miRNAs in their sequences have many common elements in their promoters for binding the same transcription factors. Moreover, by analyzing intronic miRNAs, we also obtained comprehensive evidence that miRNA-harboring introns are spliced more slowly than other introns.

**Conclusions:**

The precisely mapped transcription start sites of pri-miRNAs, and the list of transcription factors for pri-miRNAs regulation, will be valuable resources for future studies to understand the regulatory network of miRNAs.

**Electronic supplementary material:**

The online version of this article (doi:10.1186/s12864-016-3252-7) contains supplementary material, which is available to authorized users.

## Background

Most of the biological pathways in cells are influenced by microRNAs (miRNAs) [1]. Therefore, the precise regulation of miRNA expression is essential to maintain cellular homeostasis. Deviation from the wild-type expression of miRNAs results in diverse types of diseases [2, 3]. To fine-tune the expression of miRNAs, their maturation is regulated by multiple mechanisms. The regulatory steps in the miRNA biogenesis pathway include differential processing by nucleases such as DROSHA and DICER, nucleotide modification, and degradation at the intermediate step. However, miRNA expression is primarily regulated through transcriptional control [1].

The transcription of miRNA genes produces primary miRNAs (pri-miRNAs), which are used as a substrate of the DROSHA-DGCR8 protein complex. Through the action of DROSHA, the pri-miRNAs are cleaved into hairpin-like precursor miRNAs (pre-miRNAs). The pre-miRNAs serve as the substrate for DICER, which is the nuclease that produces mature miRNAs. Interestingly, the genomic location of about one-half of pri-miRNAs overlaps with other transcription units [4, 5]; moreover, in most of the cases, these pre-miRNAs sequences overlap with the introns of protein-coding genes. Many biochemical studies have confirmed that these intronic miRNAs are co-transcribed as a single transcript with the overlapping protein-coding genes, which are called the host genes of miRNAs [4, 6–8]. Accordingly, most of the intronic miRNAs share common regulatory elements for their co-expression with their host genes. It is also plausible that some miRNAs have independent promoters in specific tissues as implied by a recent bioinformatics analysis [9].

Compared to that of intronic miRNAs, the transcriptional regulation of intergenic miRNAs is not easy to understand. The primary reason for this difficulty is that the gene structure of intergenic miRNAs is not well established. In general, the genomic elements near the intergenic miRNAs, other than the location of pre-miRNA sequences, are largely unknown. Therefore, it is hard to determine the gene structure of intergenic miRNAs and the analysis of their promoter elements is not feasible.

To understand the elements in the miRNA promoters, transcription start sites of miRNAs need to be identified. Although several studies have analyzed the transcription start sites of miRNAs and the promoter elements of intergenic miRNAs, these analyses were limited due to the low resolution of the experiments [9–17]. Most of these studies inferred the transcription start sites of pri-miRNAs based on high-throughput data, such as RNA sequencing (RNA-seq) data and chromatin modification patterns. However, such high-throughput analyses were not corroborated further using biochemical assays.

**Fig. 1:**
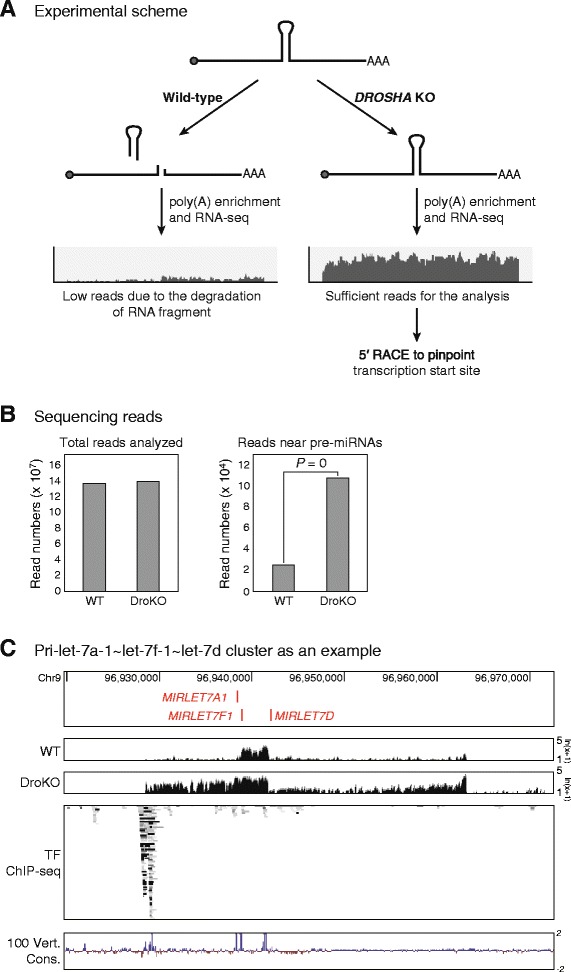
RNA sequencing of *DROSHA* knockout cells to identify the transcription start sites of human miRNAs. **a** Experimental scheme: In wild-type cells in which DROSHA protein is intact, RNA fragments made by DROSHA-mediated cleavage of pri-miRNAs, except for pre-miRNAs, are rapidly degraded (*left panel*). In *DROSHA* knockout cells, pri-miRNAs with the sequence of intact 5′ ends are accumulated (*right panel*). By analyzing the RNA sequences in these cells and verifying the 5′ end sequences through 5′ RACE, the transcription start sites can be identified precisely. **b** Total read numbers analyzed in this study, and the read numbers mapped near pre-miRNAs were shown. The enrichment of reads near pre-miRNAs in comparison to total reads were calculated by Fisher’s exact test. See Additional file 2 for details. **c** Pri-let-7a-1 ~ let-7f-1 ~ let-7d cluster as an example. Note that in comparison to the graph showing RNA signals from wild-type cells, the one representing *DROSHA* knockout cells delineates the 5′ end of pri-miRNAs more clearly. By utilizing the ChIP-seq data at the promoter region of pri-miRNAs, transcription factors that may affect the expression of pri-miRNAs can be analyzed. The ChIP-seq data was obtained from the ENCODE project [21], and vertebrate conservation data were downloaded from The USCS Genome Browser [30]

To identify the transcription start sites of miRNAs, RNA-seq data, with higher signal near the 5′ end of pri-miRNAs, is essential. Due to the continuous cleavage of pri-miRNAs by DROSHA and the rapid degradation of the remaining fragments after processing, the number of RNA fragments containing the information of the 5′ end of pri-miRNAs remains low in the cells. This makes the identification of the gene structure of pri-miRNAs difficult (Fig. 1a).

In this study, we used human *DROSHA* knockout cells to analyze the gene structure of pri-miRNAs. By analyzing the data from RNA-seq from *DROSHA* knockout cells and confirming it through rapid amplification of cDNA ends (RACE), we mapped the 5′ end of pri-miRNAs precisely. In addition, we analyzed the promoter regions of miRNA genes and identified the transcription factors that may regulate pri-miRNA expression. Our data will be very useful for future analysis of the transcriptional regulation of miRNAs.

## Results

### Exploiting *DROSHA* knockout cells to analyze intergenic miRNA gene structure

To identify the transcription start sites of intergenic miRNAs, we utilized the *DROSHA* knockout human colorectal cancer cell lines that we established recently [18]. In contrast to the cells with intact DROSHA, in which the pri-miRNAs cleaved rapidly, the pri-miRNAs in the *DROSHA* knockout cells showed increased accumulation (Additional file 1), which enabled us to identify the gene structure of intact pri-miRNAs more easily. We extracted total RNA from the *DROSHA* knockout cells and their corresponding parental wild-type cells. As pri-miRNAs are known to be polyadenylated [19], we enriched the polyadenylated RNAs and used them for next-generation sequencing (Fig. 1a).

From the analysis of the sequencing results, we confirmed that the number of reads from genomic regions encompassing miRNA hairpin sequences was highly increased in *DROSHA* knockout library compared to wild-type library (Fig. 1b and Additional file 2). Compared to the sequencing reads obtained from the library made using wild-type cells, those from the *DROSHA* knockout library delineated the transcribed region more reliably (Fig. 1c and Additional file 3). Notably, the graph representing the sequencing reads from the *DROSHA* knockout library showed a prominent boundary at the 5′ end of the transcribed region (Fig. 1c and Additional file 3). Therefore, it can be expected that pri-miRNA transcription begins at this 5′ end site and it is possible to pinpoint the transcription start site based on the RNA-seq data from *DROSHA* knockout cells.

### Precise mapping of the transcription start sites of miRNAs

**Fig. 2:**
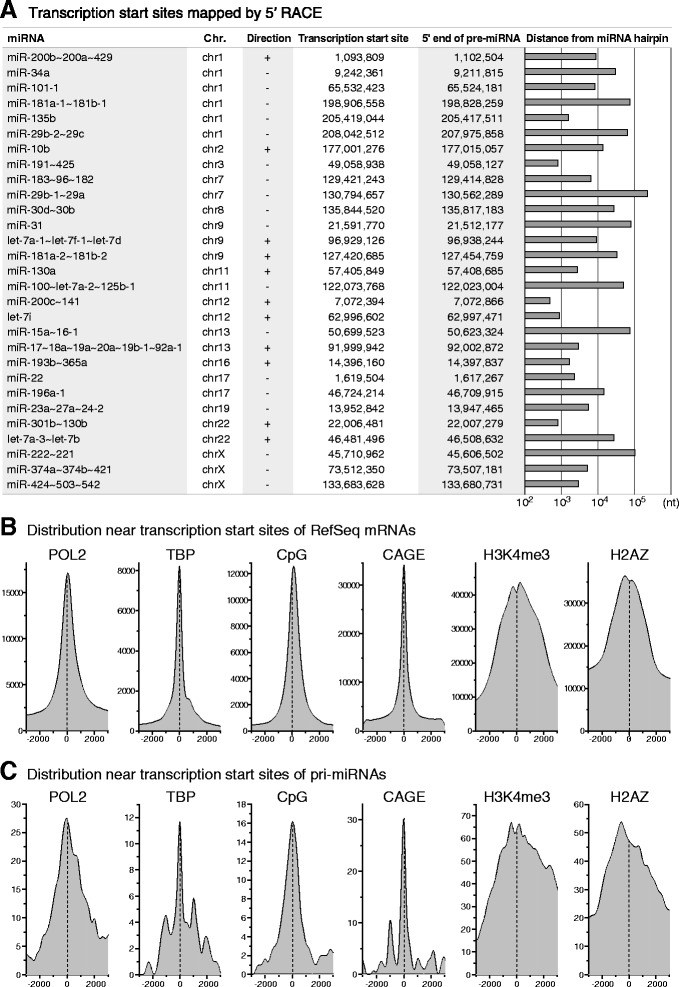
Analysis of the transcription start sites of miRNAs. **a** Transcription start sites mapped by 5′ RACE. Based on the data from 5′ RACE, the transcription start sites of pri-miRNAs (GRCh37/hg19) were annotated. The 5′ end positions of pre-miRNAs were identified by manual inspection of sequencing data of mature miRNAs from HCT116 cells [18]. Distances between the transcription start sites and miRNA hairpin sequences were calculated and shown as a bar graph. **b** and **c** Distribution of Pol II, TBP, CpG islands, CAGE tags, H3K4me3, and H2A.Z, near the transcription start sites of (**b**) mRNAs and (**c**) miRNAs. The ChIP-seq data of Pol II, TBP, H3K4me3, and H2A.Z, and the information of CAGE tags were obtained from the ENCODE project [21]. In the case that the data for HCT116 cell line is not available, the data for other solid cancer cell lines, including A549, HepG2, and MCF7, were mixed and used for analysis. The information of CpG islands was obtained from The UCSC Genome Browser [30]. The distribution of these factors near the 5′ ends of mRNAs was analyzed using the information of 5′ end positions of RefSeq mRNAs from The UCSC Genome Browser [30]. The graph has been depicted with a bin of 200 nts

To pinpoint the transcription start site of pri-miRNAs, we employed the RACE technique; to select miRNA candidates for RACE, we applied several criteria. First, we selected candidates among 274 miRNAs that are included in the list of authentic miRNAs, which we had reported previously [20]. Briefly, we carefully selected these authentic miRNAs from the whole miRBase entries, by observing their expressions from a large number of sequencing libraries, analyzing the homogeneity at 5′ termini of their sequences, and curating them through manual inspection based on literature; therefore, they are expected to be biologically important. These miRNAs comprise 175 pri-miRNAs, since clustered miRNAs in close genomic proximity are transcribed as a single transcript (Additional file 4). The co-transcription of miRNA clusters that we selected for RACE was confirmed by literature search, expressed sequence tags (ESTs) analysis, or PCR experiments (Additional files 4 and 5). Second, by inspecting the graphs of sequencing reads (Fig. 1c and Additional file 3) we selected only those pri-miRNAs whose expression signals were detected at high levels in the HCT116 cells that we tested (see Methods). In addition, only the pri-miRNAs whose reads were enriched in *DROSHA* knockout library compared to wild-type library were selected. However, the pri-miRNAs that overlap with protein-coding genes in the genome were excluded from this inspection. After filtering, 34 pri-miRNAs that met our criteria were selected (Additional file 4). When we compared the graphs of sequencing reads of the candidate pri-miRNAs between *DROSHA* knockout and wild-type libraries, most of the pri-miRNAs showed enrichment of reads in the regions containing pre-miRNA hairpin sequences (Fig. 1c and Additional files 3 and 4). We assumed that these enriched regions were the transcribed regions of pri-miRNAs and designed PCR primers for their 5′ end cloning. By performing 5′ RACE, we could pinpoint the 5′ ends of 29 pri-miRNAs, which account for 60 mature miRNAs. We annotated these 5′ end nucleotides as the transcription start sites of pri-miRNAs (Fig. 2a).

The calculated distances between the identified transcription start sites and the 5′ end nucleotides of pre-miRNAs sequences ranged from less than 1 kilobase (kb) to more than 200 kilobases (kbs). For example, the transcription of miR-200c, which is a member of the pri-miR-200c ~ 141 cluster (tilde indicates transcript spanning the indicated gene cluster), is initiated 472 base pairs (bps) upstream of the hairpin sequence. The genomic distances between the transcription start sites and pre-miRNA hairpins for 16 out of 29 pri-miRNAs are less than 10 kbs (Fig. 2a). In some cases, however, the transcription start site was identified more than 100 kbs upstream of the pre-miRNA sequences (pri-miR-222 ~ 221 and pri-miR-29b-1 ~ 29a). We could not identify any factor that might affect the distances between the transcription start sites and the position of pre-miRNAs.

We analyzed the distribution of RNA polymerase II (Pol II) and TATA-binding protein (TBP) signals near the transcription start sites of pri-miRNAs using the public data from the Encyclopedia of DNA Elements (ENCODE) project [21]. As a control, we compared the distribution to that of Pol II and TBP signals near the transcription start sites annotated for the RefSeq mRNAs. The distribution graphs of Pol II and TBP were similar between pri-miRNAs and RefSeq mRNAs with the signals made a peak near the transcription start site (Fig. 2b and c). This suggests that Pol II and TBP bind near the transcription start sites of pri-miRNAs, similar to the case in mRNAs, and initiate transcription from this region in consistent with our RACE results. Moreover, the transcription start sites of pri-miRNAs clearly associated with CpG islands in a similar pattern with that of RefSeq mRNAs. We also observed the cap analysis gene expression (CAGE) data near transcription start sites of pri-miRNAs and RefSeq mRNAs, and found that the peak of CAGE signals exactly matched with the transcription start sites in both cases (Fig. 2b and c) [21]. The promoter-associated histone modifications including H3K4me3 and H2A.Z were also enriched near the transcription start sites of miRNAs. Overall, these data show that the pri-miRNAs analyzed in this study actually initiate their transcription from the positions obtained from our RACE analysis.

### Analysis of transcription factor binding to miRNA promoters

Although the regulatory relationship between miRNAs and their target mRNAs has been studied extensively, the transcriptional regulation of miRNAs themselves by transcription factors is not well understood. To analyze the transcriptional regulation of miRNAs, we utilized the data generated by the ENCODE project, which contains the chromatin immunoprecipitation followed by sequencing (ChIP-seq) data for 161 transcription factors [21]. To identify the transcription factors that may regulate miRNA expression, we selected the genomic region spanning the promoter of the pri-miRNA, from −2000 to +500 (i.e., 2000 nts upstream to 500 nts downstream of the transcription start site) as the binding region of transcription factors. Although some transcription factors regulate transcription from a distance, most proximal binding events of functional transcription factors can be captured in this region. Consistent with this notion, the binding sites of transcription factors have been found to be concentrated in this region [22–25]. After collecting promoter sequences of pri-miRNAs, we looked for ChIP-seq signals for transcription factors within this region. As a control, we collected the sequences in corresponding regions near the transcription start sites of RefSeq mRNAs.

**Fig. 3:**
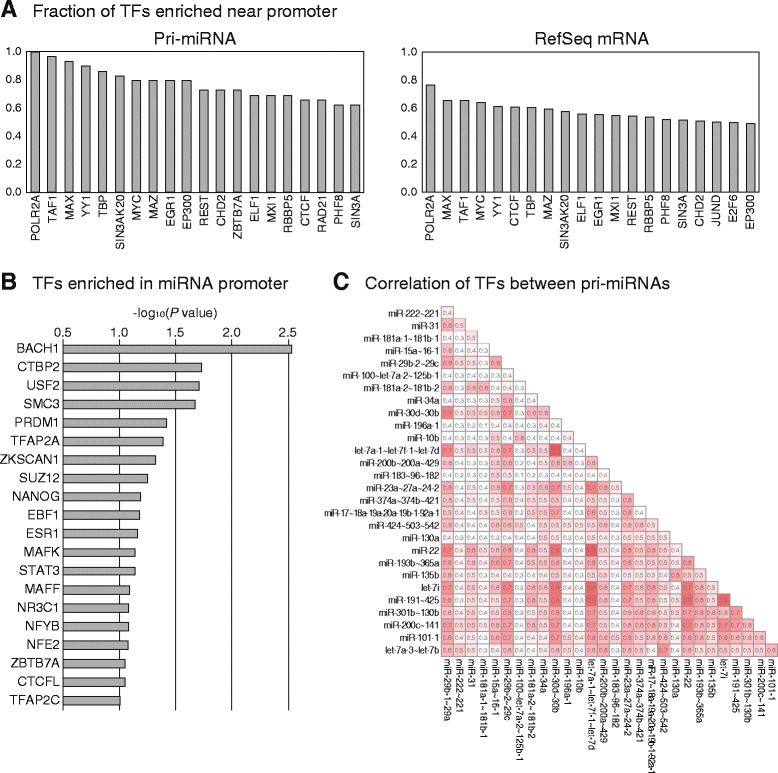
Analysis of transcription factor binding to the promoters of miRNA genes. **a** The fraction of transcription factors enriched near the promoters of miRNA and mRNA genes, respectively, are shown. Twenty (out of 161) transcription factors that show high binding preference for miRNA or mRNA promoters, are shown. **b** Transcription factors more enriched in the promoters of miRNAs than in those of mRNAs, are shown. The transcription factors with *P* value less than 0.1, are shown. *P* value was calculated by Fisher’s exact test. **c** Correlation of transcription factors between pri-miRNA pairs. For each pri-miRNA pair, we calculated the fraction of the transcription factors that commonly bind to the promoters of both pri-miRNAs (see Methods)

We found the binding sites of diverse transcription factors to be clustered in the promoter region of pri-miRNAs that we selected (Figs. 1c and 3a). Notably, the transcription start sites of all selected pri-miRNAs contained the ChIP-seq signal for Pol II (POLR2A) in this region. In contrast, about a quarter of the promoters of RefSeq mRNAs did not contain the ChIP-seq signal for Pol II (Fig. 3a). In addition to Pol II, other general transcription factors, such as TBP and TAF1, also showed a higher fraction of binding to pri-miRNA promoters than to RefSeq mRNA promoters. It is possible that the RefSeq mRNAs without ChIP-seq signal for general transcription factors at their promoters might be expressed at lower levels in the cells. When we divided the RefSeq mRNAs into four classes based on their expression levels, the group of highly expressed mRNAs also associated with the general transcription factors at a higher degree (Additional file 6).

Upon comparing the binding fractions of transcription factors between the pri-miRNA and RefSeq mRNA promoters, we found that several transcription factors were significantly enriched in pri-mRNA promoters (Fig. 3b). Among the highly enriched factors, there were many that were functionally related; for example, the transcription factor MAFF was found to bind to the promoters of miRNAs to which another transcription factor MAFK also bound (Additional file 7). Based on this data and previous reports that showed MAFF and MAFK to be members of the same protein family, we suggest that they may work together to regulate the expression of common miRNAs [26]. Interestingly, several transcription factors including TFAP2A, EBF1, and STAT3, were also included in the list of enriched transcription factors for intergenic miRNAs from a previous study [9].

It is plausible that a similar set of transcription factors would bind to the promoters of miRNAs that need to be regulated together. To identify such pairs of miRNAs, which might be regulated by common transcription factors, we calculated the correlation value between transcription factor binding to promoters of both the pri-miRNAs of each pair; these correlation values differed considerably among pri-miRNA pairs (Fig. 3c, see Methods). The correlation values between paralogous pri-miRNAs, i.e., between pri-miR-181a-1 ~ 181b-1 and pri-miR-181a-2 ~ 181b-2, or between pri-miR-29b-1 ~ 29a and pri-miR-29b-2 ~ 29c, were high (0.60 and 0.64 respectively), as expected. It is possible that they are under the control of similar sets of transcription factors because they had originated from the same ancestor gene. Interestingly, some miRNA pairs, which did not have any sequence homology, showed higher correlation values than did paralogous pri-miRNAs pairs; for example, pri-let-7a-1 ~ let-7f-1 ~ let-7d and pri-miR-30d ~ 30b showed correlation value of 0.83, which suggests that these pri-miRNAs are under the transcriptional control of a highly overlapping set of transcription factors (Fig. 3c). To test whether the high correlation of transcription factor binding to pri-miRNA promoters results in a high correlation of the expression levels of the corresponding mature miRNAs, we selected the miRNA pairs from the top 20 % and the bottom 20 % of the list of pri-miRNA pairs placed in the order of their correlation values for transcription factor binding (Fig. 3c). We compared the correlation of each pri-miRNA pair with the correlation between the expression of the corresponding mature miRNAs, which was determined using the expression profiles of mature miRNAs from diverse tissues [27]. Interestingly, the pri-miRNA pairs from the top 20 % of the list also showed higher correlation between the expression of their mature miRNAs (Additional file 8), suggesting that they are indeed under similar transcriptional control; as a result, the mature miRNAs tend to be expressed simultaneously from these pri-miRNAs.

### Expression analysis of the host genes of intronic miRNAs

**Fig. 4:**
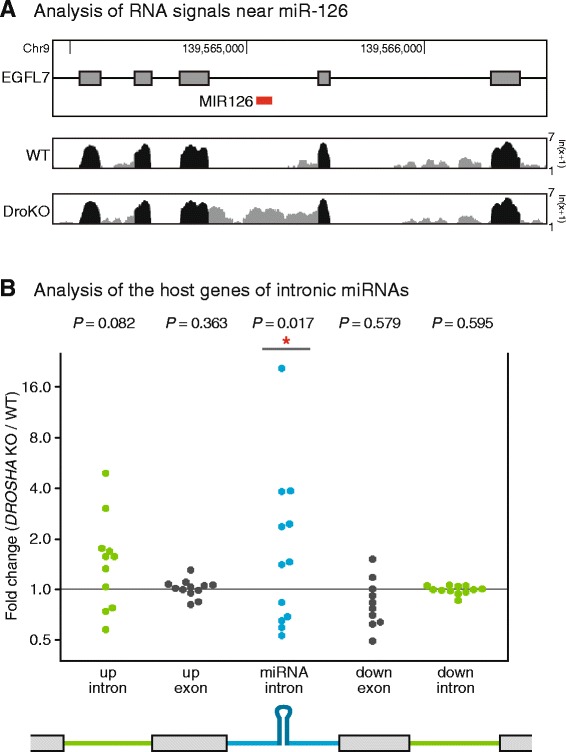
Analysis of the host genes of intronic miRNAs. **a** Analysis of the sequencing graph encompassing *EGFL7* gene, as an example. A part of the *EGFL7* gene encompassing pre-miR-126, is shown. In the sequencing graph, exonic and intronic reads are depicted by *black* and *gray* colors, respectively. Note that only the RNA signals at the intron containing the pre-miR-126 are enriched in the *DROSHA* knockout cells. **b** Analysis of the host genes of intronic miRNAs. Among the authentic miRNAs [20], intronic miRNAs were selected for analysis (Additional file 4). Fold change of the read numbers corresponding to each genomic region was calculated (*DROSHA* knockout / wild-type cells). *P* value was calculated using two-sided paired t-test (* < 0.05)

In the case of intergenic miRNAs, most of the regions covering pri-miRNA sequences were enriched by *DROSHA* ablation (Fig. 1c and Additional file 3). Surprisingly, however, analysis of sequencing reads of the host genes of intronic miRNAs showed an increase in the number of sequencing reads of only the introns that harbor miRNA hairpin sequences. For example, analysis of the genomic locus of *EGFL7* gene, which is the host gene of a well-known intronic miRNA, miR-126, showed an increase in the number of sequencing reads of only the miRNA-containing intron from *DROSHA* knockout cells, compared to those from wild-type cells (Fig. 4a and Additional file 9). None of the other introns and exons showed a significant difference between wild-type and *DROSHA* knockout cell lines. These observations confirm our previous results that the splicing of only the miRNA-containing introns is delayed by DROSHA knockdown, while the production of mature mRNAs is not affected [4].

To determine whether this result applies to other intronic miRNAs, we analyzed the host genes of all intronic miRNAs that show detectable expression in the cells that we tested (Additional file 4). For most intronic miRNAs, we found a significant enrichment of sequencing reads of the miRNA-containing introns, although in some cases, this enrichment was not seen (Fig. 4b and Additional file 10). These results suggest that our previous observation can be generalized for most intronic miRNAs [4]. We also noted the enrichment of sequencing reads of the neighboring upstream intron, for some intronic miRNAs, albeit less significant. We do not understand the underlying mechanism of this phenomenon; however, one possible explanation is that the upper intron is also influenced by the protein complexes bound to the miRNA hairpin-containing intron. Interestingly, we found that the sizes of miRNA-containing introns with enriched reads in *DROSHA* knockout compared to wild-type, are slightly but significantly shorter than the sizes of miRNA-containing introns without reads enrichment (Additional file 11). In addition, the relative positions of miRNA-containing introns with enriched reads had a tendency to be located more downstream of host genes (close to 3′ end of genes). Further analysis is required to understand the crosstalk between miRNA processing and splicing reactions.

## Discussion

Several studies have investigated the structure of miRNA genes and analyzed miRNA promoters. One of the recent studies used sequencing data from *Drosha* knockout mouse embryonic stem cells to validate the algorithm for the prediction of transcription start sites of miRNA genes [15]. In this study, in comparison to previous studies, more accurate prediction of transcription start sites of pri-miRNAs was possible, using the information about the transcribed region covering pri-miRNA sequences from *Drosha* knockout cells. However, the transcription start sites were assigned based only on computational analysis, and were not confirmed further through biochemical assays. Another recent study used a transdominant negative DROSHA (TN-DROSHA) to hinder the processing of pri-miRNAs, thereby increasing the amount of pri-miRNAs in the cells [16]. The treatment with TN-DROSHA enriched the sequencing reads from transcribed regions of pri-miRNAs, which enabled the identification of novel structures in the pri-miRNA transcripts [16]. Based on computational re-construction of pri-miRNA transcripts, it was shown that many miRNAs could be regulated by tissue-specific manner through alternative promoters. Compared to this setting, where functional DROSHA proteins still can process pri-miRNAs in the TN-DROSHA-treated cells [28], the use of *DROSHA* knockout cells can enrich the transcribed regions of pri-miRNAs more strongly because no DROSHA activity exists in the cells.

The *DROSHA* knockout cells that we used in this study offer several advantages for the identification of pri-miRNA gene structure because the processing of canonical pri-miRNAs is completely abolished in this cell line. By using the *DROSHA* knockout human cells, we could obtain more in-depth information about the transcribed regions of human pri-miRNAs with more reliability. Importantly, we confirmed the RNA-seq data through 5′ RACE, which enabled us to identify the transcription start sites of pri-miRNAs at the nucleotide level. This study is the first one to confirm the transcription start sites of many intergenic miRNAs using biochemical assays.

The analysis of the promoters of intergenic miRNAs shows that there are several transcription factors that prefer to bind to the promoters of miRNAs over those of RefSeq mRNAs (Fig. 3b). Although the physiological consequence of miRNA regulation by these transcription factors is largely unknown currently, its elucidation will help us to understand the complex cellular regulatory network in the future.

The RNA-seq data of *DROSHA* knockout cells reported in this study will be a valuable resource for future studies. For example, this data can be used to identify transcriptions start sites of other pri-miRNAs not included in this study. In addition, this data will be helpful to understand the transcript structure of pri-miRNAs, such as the analysis of splicing patterns or transcription termination sites.

## Conclusions

From this study, we identified the transcription start sites of pri-miRNAs precisely by analyzing the RNA-seq data from *DROSHA* knockout cells and confirming it through RACE experiments. We also identified transcription factors for pri-miRNAs regulation. These data will be valuable resources for future studies to understand the regulatory network of miRNAs.

## Methods

### Cell culture

The colorectal cancer cell line, HCT116 (Korean Cell Line Bank), was maintained in McCoy’s 5A medium supplemented with 10 % fetal bovine serum (WelGENE). The *DROSHA* knockout cell line was established, as described previously [18].

### RNA sequencing and analysis

TRIzol reagent (Life Technologies) was used to extract total RNA from wild-type or *DROSHA* knockout HCT116 cells. To enrich pri-miRNAs, which contain poly(A) tails, oligo(dT) Dynabeads (Life Technologies) was used. The sequencing library was made using TruSeq Stranded Total RNA Library Prep Kit (Illumina), and the quality of the library was checked using Agilent 2100 Bioanalyzer (Agilent). Finally, the library was sequenced on HiSeq 2500 (Illumina) for 50 sequencing cycles. FASTQ sequences obtained from the sequencer were trimmed to remove nucleotides with lower quality values. And then, the sequences were aligned into the human genome using Bowtie2 algorithm with default parameters [29]. Among the aligned reads, only the reads with perfect match and those with mapping quality greater than 10 were remained. The command line is as followed.

fastx_trimmer -Q 33 -f 2 -l 37 -i seq.fastq | bowtie2 -x ./hg19 -q - | grep "^@\|XM:i:0" - | samtools view -q 10 -Sb - > aligned.bam

After converting the results into bedGraph files, the graphs representing RNA signals of wild-type and *DROSHA* knockout cells were compared. For the selection of pri-miRNA candidates of RACE analysis, we examined the signals between predicted transcription start sites of pri-miRNAs and 5′ end of pre-miRNAs. We selected the pri-miRNAs whose peaks in bedGraph are higher than 10. In addition, the pri-miRNAs whose peaks in the *DROSHA* knockout library are more than 50 % higher than those in the wild-type library were selected.

### Rapid amplification of cDNA ends (RACE)

The generation of cDNA for 5′ RACE was carried out according to the manufacturer’s protocol (GeneRacer Kit, Invitrogen). From the analysis of transcribed region based on the RNA-seq data, we designed primers to amplify the 5′ end region of pri-miRNAs. The sequences of primers are included in the Additional file 12. After cloning the amplified fragments, we analyzed their sequences by Sanger sequencing. For each selected miRNA, we chose the clones with the same 5′ end sequence, which represent more than 50 % of all the sequenced clones, and annotated this 5′ end sequence as the transcription start site for the pri-miRNA.

### Analysis of transcription factor binding to miRNA promoters

We assumed the promoter of miRNAs to be the region from 2000 nts upstream to 500 nts downstream from the transcription start site of pri-miRNAs. To analyze the binding of transcription factors to promoters of intergenic miRNAs, we downloaded the genomic coordinates of the ChIP-Seq data of transcription factors generated by the ENCODE project [21]. By comparing the coordinates of promoter regions of miRNAs with those of transcription factors, we obtained the list of transcription factors with binding sites for the promoter of each miRNA (Additional file 7). To calculate the correlation value between pri-miRNAs pair (miR-A and miR-B) in Fig. 3c, following formula was used.

Correlation value = (Number of transcription factors common for both miR-A and miR-B) / ((Number of transcription factors for miR-A) X (Number of transcription factors for miR-B))^0.5^

### Analysis of intronic miRNAs

The genomic coordinates of introns and exons of each host gene having intronic miRNAs were obtained from The UCSC Genome Browser (http://genome.ucsc.edu/). For each intron or exon, the number of sequencing reads from wild-type and *DROSHA* knockout cells were counted. If the read number for an intron or an exon was too low (<10), that region was excluded from further analysis. After this filtering, the fold changes in read numbers (*DROSHA* knockout/wild-type) for each region was calculated.

## Additional files

Additional file 1: Measurement of the level of pri-miRNAs in *DROSHA* knockout cells. To amplify pri-miRNAs, TaqMan Pri-miRNA assay kit (Applied Biosystems) was used. The amount of pri-miRNAs was normalized against that of U6 small nuclear RNA, and compared between wild-type and *DROSHA* knockout cells. Error bar shows the standard error from three independent samples (*n* = 3). (PDF 84 kb)
Additional file 2: Summary of sequencing results. Total reads obtained from sequencer and statistics of aligned reads were shown. We only used the aligned reads with mapping quality greater than 10 (see Methods). For the analysis of read numbers near pre-miRNAs, those reads with the distances from pre-miRNAs are closer than 500 nts were counted. *P* value was calculated by Fisher’s exact test. (PDF 136 kb)
Additional file 3: Six representative pri-miRNAs with the graphs showing RNA signals from wild-type and *DROSHA* knockout cells were shown as in the Fig. 1c. (PDF 170 kb)
Additional file 4: List of pri-miRNAs analyzed in this study. From the list of authentic miRNAs, which were established previously [20], the list of pri-miRNAs was generated based on the genomic locations of mature miRNAs. The pri-miRNAs were divided into two groups (A and B) depending on their overlap with protein-coding genes. By inspecting the RNA-seq data, the signal intensity was annotated as either ‘High’, ‘Low’, or ‘No’ (see Methods). (A) For intergenic pri-miRNAs with no overlap with protein-coding genes, the enrichment of RNA signals in the *DROSHA* knockout cells, compared to those in wild-type cells, was determined. Thirty-four pri-miRNAs that met our criteria are shown with bold letters. For the clustered miRNAs among the pri-miRNAs analyzed by RACE, the evidence of co-transcription was indicated with citations for 11 out of 20 clustered miRNAs. For six pri-miRNAs, we identified the expressed sequence tags (ESTs) with sequences spanning all miRNA members of each cluster and included the GenBank IDs of representative ESTs. For the remaining 3 miRNA clusters, we performed PCR experiments to confirm that the transcripts spanning all miRNA members are increased in the cDNA made from *DROSHA* knockout cells compared to that from wild-type cells. We included the results into Additional file 5. (B) For intronic miRNAs, which reside inside the introns of protein-coding genes, the accumulation of RNA signals at the miRNA-containing intron in the *DROSHA* knockout cells, compared to that in wild-type cells, was determined. (XLSX 17 kb)
Additional file 5: The confirmation of co-transcription of miRNA clusters. PCR was performed to amplify the region spanning miRNA members of each pri-miRNA. The expression of each amplicon was measured using the cDNA made from wild-type and *DROSHA* knockout cells. (PDF 196 kb)
Additional file 6: The fraction of transcription factors near the promoters of mRNA genes. Based on the expression level, mRNAs were divided into four groups, and the fractions of transcription factors were calculated at each group, respectively, as in the Fig. 3a. (PDF 86 kb)
Additional file 7: List of transcription factors that may regulate the transcription of pri-miRNAs. Using the ChIP-seq data of 161 transcription factors produced by the ENCODE project [21], the binding region of each transcription factor was compared with the promoter regions of miRNAs. The transcription factors with binding sites in the promoters of miRNAs are summarized. (XLSX 15 kb)
Additional file 8: Correlation between the expressions of mature miRNA pairs. The pri-miRNA pairs from the top 20 % and the bottom 20 % of the list from Fig. 3c, placed in the order of their correlation values (from Fig. 3c), were collected. For each pri-miRNA pair, the correlation between the expression of their mature miRNAs was calculated based on the miRNA expression profiles published previously [27]. The distribution of correlation values is shown in the box plot. The *P* value was calculated by one-tailed t-test. (PDF 96 kb)
Additional file 9: Four representative intronic miRNAs and their host genes were shown with the graphs depicting RNA signals from wild-type and *DROSHA* knockout cells as in the Fig. 4a. (PDF 150 kb)
Additional file 10: The fold change values (*DROSHA* knockout / wild-type) of read counts for each exonic/intronic region of host genes of intronic miRNAs. (XLSX 12 kb)
Additional file 11: The analyses of size and genomic location of miRNA-containing introns. (A) The size of each intron/exon was compared between the host genes with enriched reads at miRNA-containing introns and those without enriched reads. (B) The relative positions of miRNA-containing introns were also compared. *P* values were calculated by Wilcoxon rank-sum test. (PDF 121 kb)
Additional file 12: The sequences of primers used for RACE experiments. The insets depict primer-binding regions, transcription start sites of pri-miRNAs analyzed from RACE experiments, and transcription termination sites expected from RNA-seq signals. The frequencies of 5′ RACE clones were also included. (XLSX 383 kb)

## Abbreviations

bp: Base pair
CAGE: Cap analysis gene expression
ChIP-seq: Chromatin immunoprecipitation followed by sequencing
ENCODE: Encyclopedia of DNA elements
EST: Expressed sequence tag
kb: Kilobase
miRNA: microRNA
Pol II: RNA polymerase II
pre-miRNA: Precursor miRNA
pri-miRNA: Primary miRNA
RACE: Rapid amplification of cDNA ends
RNA-seq: RNA sequencing
TBP: TATA-binding protein
TN-DROSHA: Transdominant negative DROSHA
