# Supplementary figures and images for: Precise mapping of the transcription start sites of human microRNAs using DROSHA knockout cells
Source: BMC Genomics. 2016 Nov 11;17:908. doi: 10.1186/s12864-016-3252-7 (PMC5106785; doi:10.1186/s12864-016-3252-7)

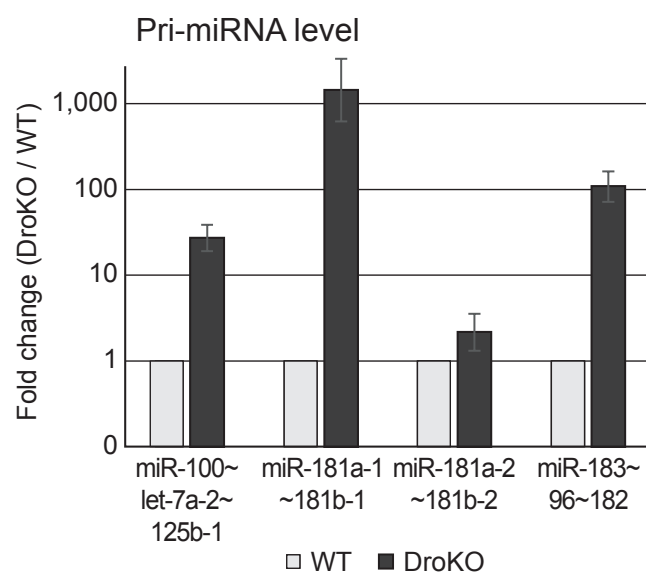

Supplement: Additional file 1: — Measurement of the level of pri-miRNAs in DROSHA knockout cells. To amplify pri-miRNAs, TaqMan Pri-miRNA assay kit (Applied Biosystems) was used. The amount of pri-miRNAs was normalized against that of U6 small nuclear RNA, and compared between wild-type and DROSHA knockout cells. Error bar shows the standard error from three independent samples (n = 3). (PDF 84 kb) [file 12864_2016_3252_MOESM1_ESM.pdf]

## A Pri-let-7i

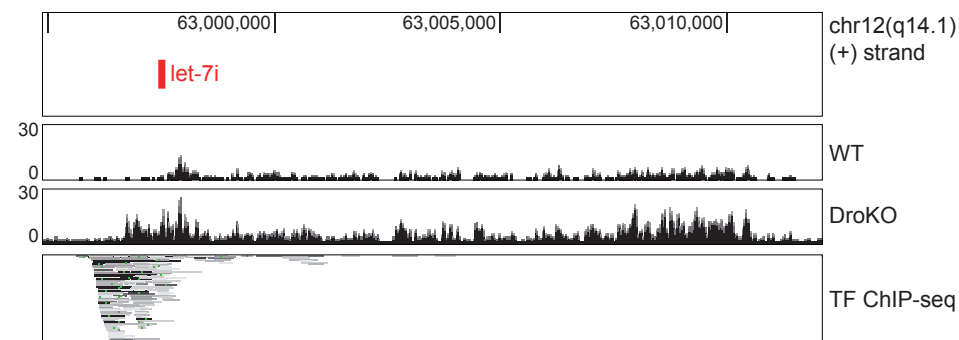

## B Pri-miR-183~96~182

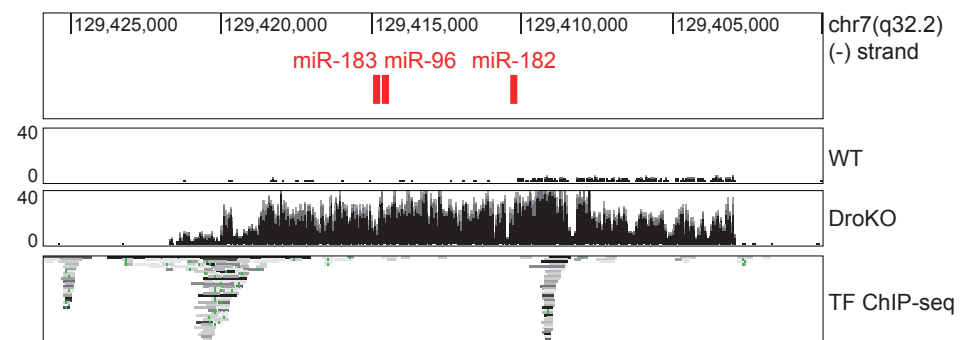

## C Pri-miR-30d~30b

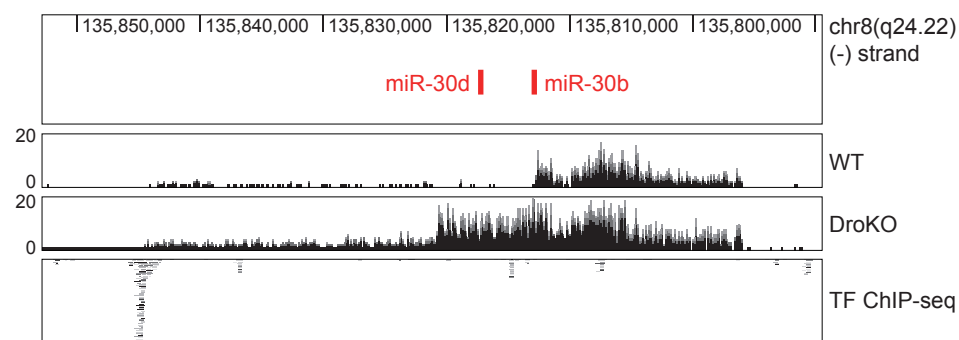

## D Pri-miR-374a~374b~421

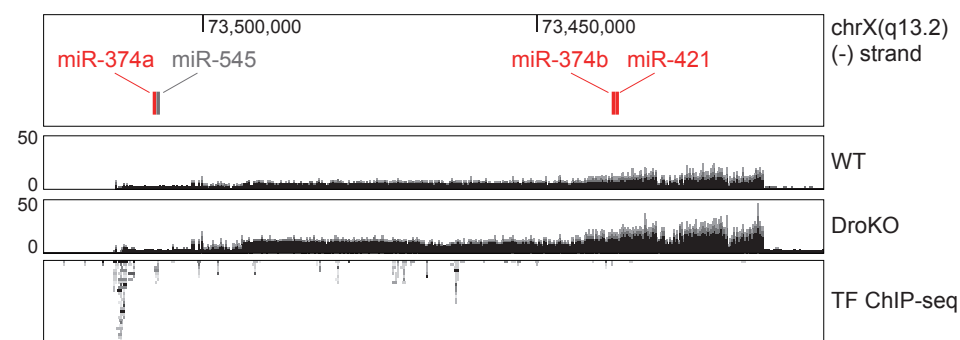

## E Pri-miR-222~221

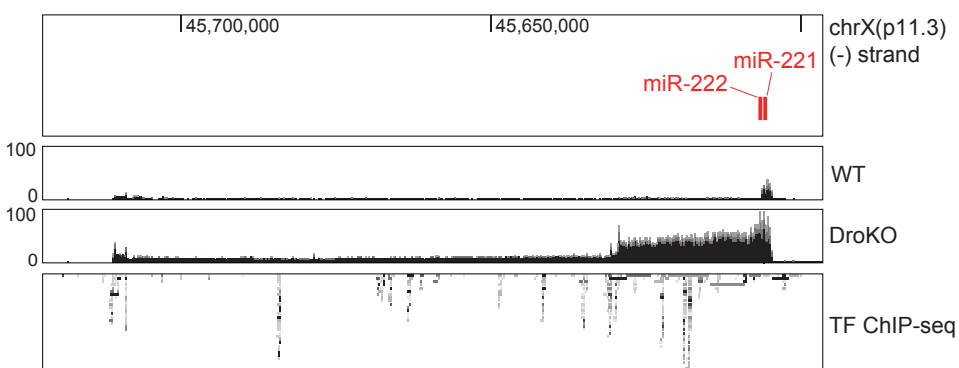

## F Pri-miR-31

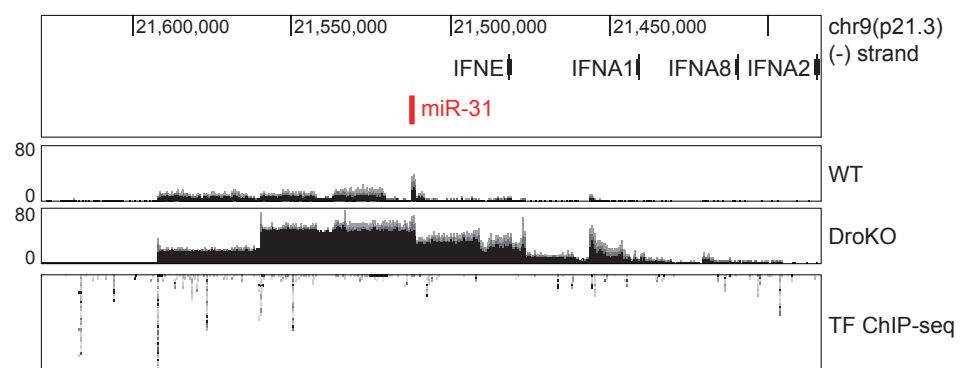

Supplement: Additional file 3: — Six representative pri-miRNAs with the graphs showing RNA signals from wild-type and DROSHA knockout cells were shown as in the Fig. 1c. (PDF 170 kb) [file 12864_2016_3252_MOESM3_ESM.pdf]

**A** Pri-miR-200b~200a~429

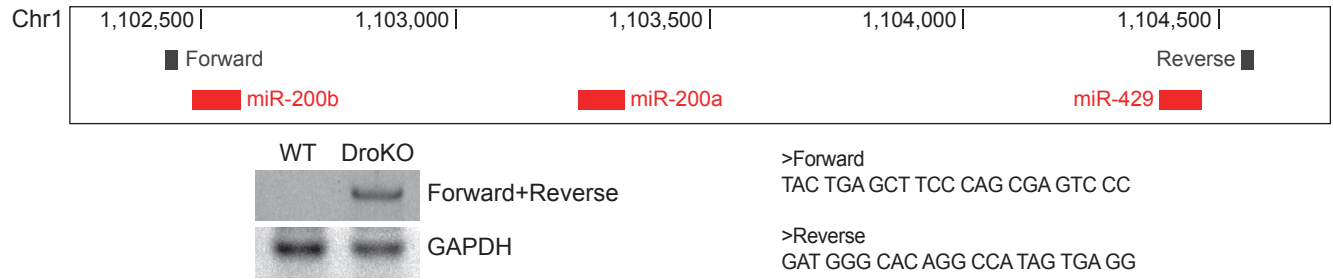

**B** Pri-miR-301b~130b

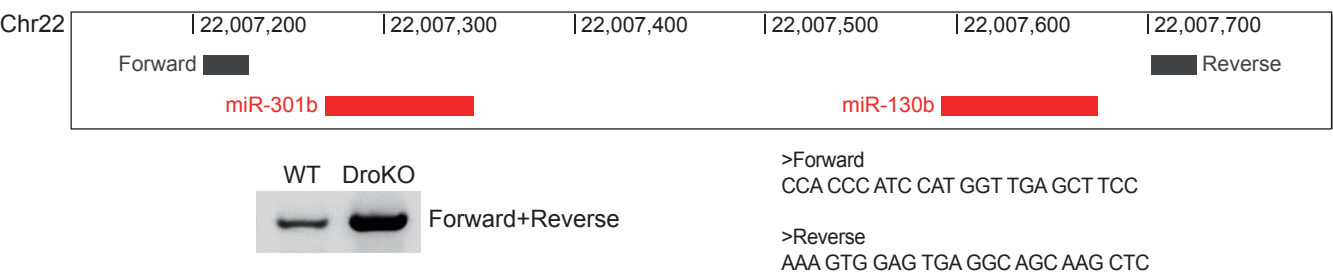

**C** Pri-miR-30d~30b

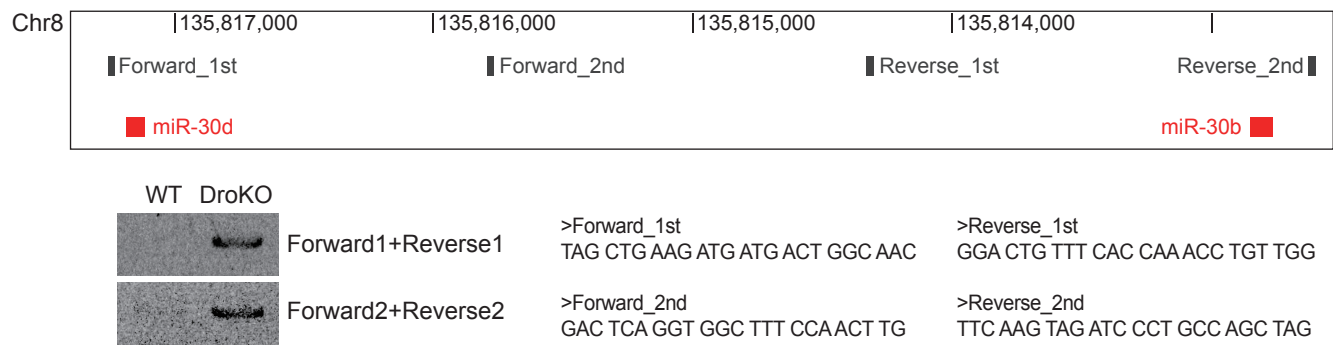

Supplement: Additional file 5: — The confirmation of co-transcription of miRNA clusters. PCR was performed to amplify the region spanning miRNA members of each pri-miRNA. The expression of each amplicon was measured using the cDNA made from wild-type and DROSHA knockout cells. (PDF 196 kb) [file 12864_2016_3252_MOESM5_ESM.pdf]

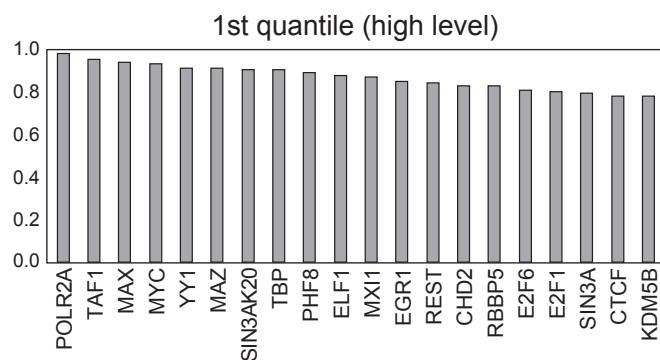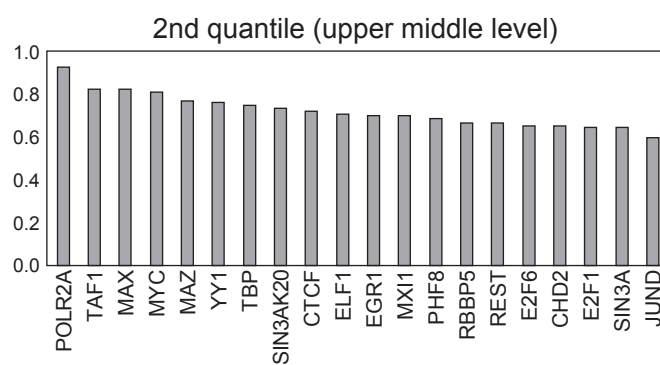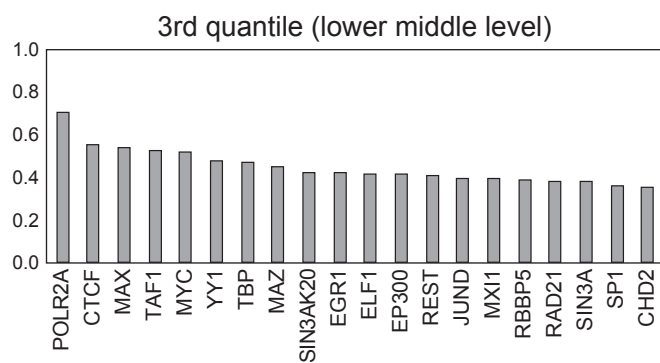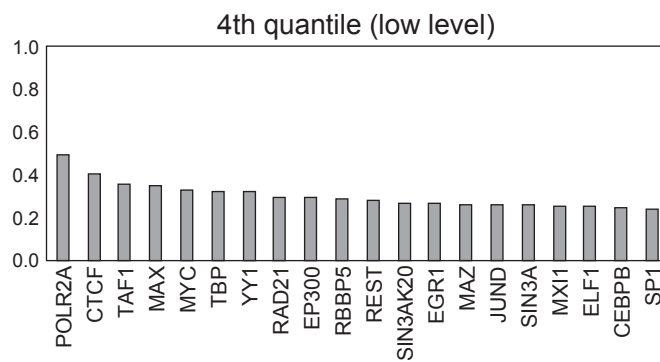

Supplement: Additional file 6: — The fraction of transcription factors near the promoters of mRNA genes. Based on the expression level, mRNAs were divided into four groups, and the fractions of transcription factors were calculated at each group, respectively, as in the Fig. 3a. (PDF 86 kb) [file 12864_2016_3252_MOESM6_ESM.pdf]

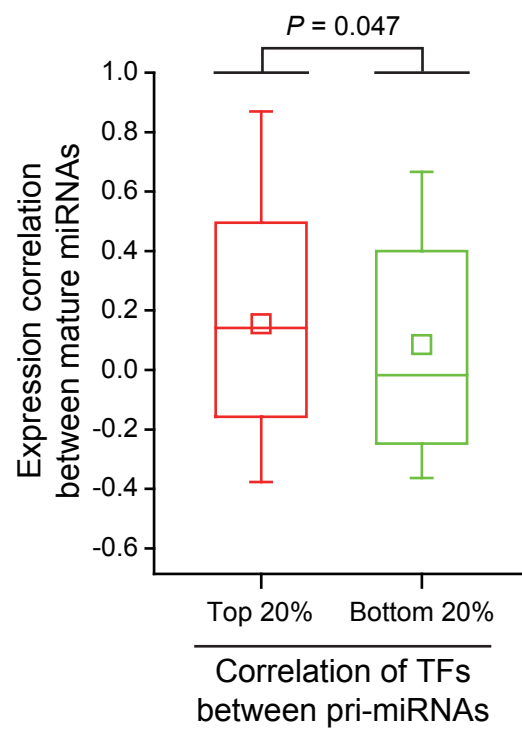

Supplement: Additional file 8: — Correlation between the expressions of mature miRNA pairs. The pri-miRNA pairs from the top 20 % and the bottom 20 % of the list from Fig. 3c, placed in the order of their correlation values (from Fig. 3c), were collected. For each pri-miRNA pair, the correlation between the expression of their mature miRNAs was calculated based on the miRNA expression profiles published previously [27]. The distribution of correlation values is shown in the box plot. The P value was calculated by one-tailed t-test. (PDF 96 kb) [file 12864_2016_3252_MOESM8_ESM.pdf]
