# Supplementary material for: Precise mapping of the transcription start sites of human microRNAs using DROSHA knockout cells
Source: BMC Genomics. 2016 Nov 11;17:908. doi: 10.1186/s12864-016-3252-7 (PMC5106785; doi:10.1186/s12864-016-3252-7)

Library	Wild-type	*DROSHA* KO	
Total reads	198,937,194	196,007,170	
Aligned 1 time	123,306,296	128,857,225	
Aligned >1 times	63,811,001	55,447,581	
Used for this study (MAPQ>10)	137,085,295	139,925,320	
Mapped near pre-miRNA	**25,514**	**108,805**	***P* value = 0**

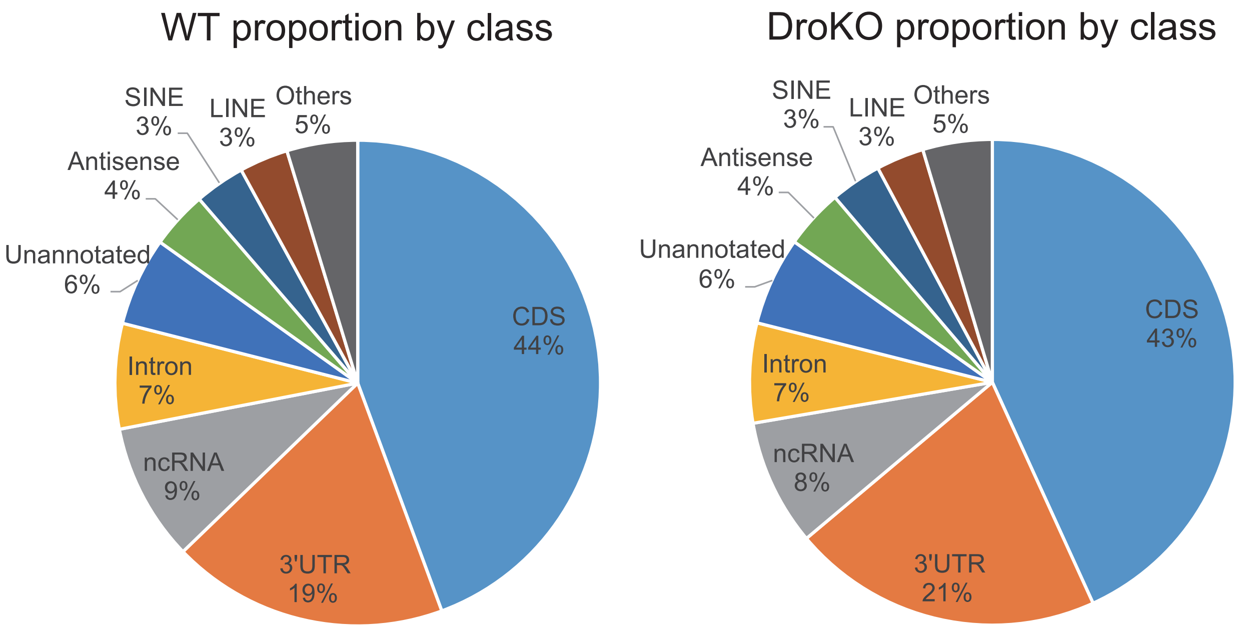

Supplement: Additional file 2: — Summary of sequencing results. Total reads obtained from sequencer and statistics of aligned reads were shown. We only used the aligned reads with mapping quality greater than 10 (see Methods). For the analysis of read numbers near pre-miRNAs, those reads with the distances from pre-miRNAs are closer than 500 nts were counted. P value was calculated by Fisher’s exact test. (PDF 136 kb) [file 12864_2016_3252_MOESM2_ESM.pdf]
