# Supplementary material for: Precise mapping of the transcription start sites of human microRNAs using DROSHA knockout cells
Source: BMC Genomics. 2016 Nov 11;17:908. doi: 10.1186/s12864-016-3252-7 (PMC5106785; doi:10.1186/s12864-016-3252-7)
Supplement: Additional file 9: — Four representative intronic miRNAs and their host genes were shown with the graphs depicting RNA signals from wild-type and DROSHA knockout cells as in the Fig. 4a. (PDF 150 kb) [file 12864_2016_3252_MOESM9_ESM.pdf]

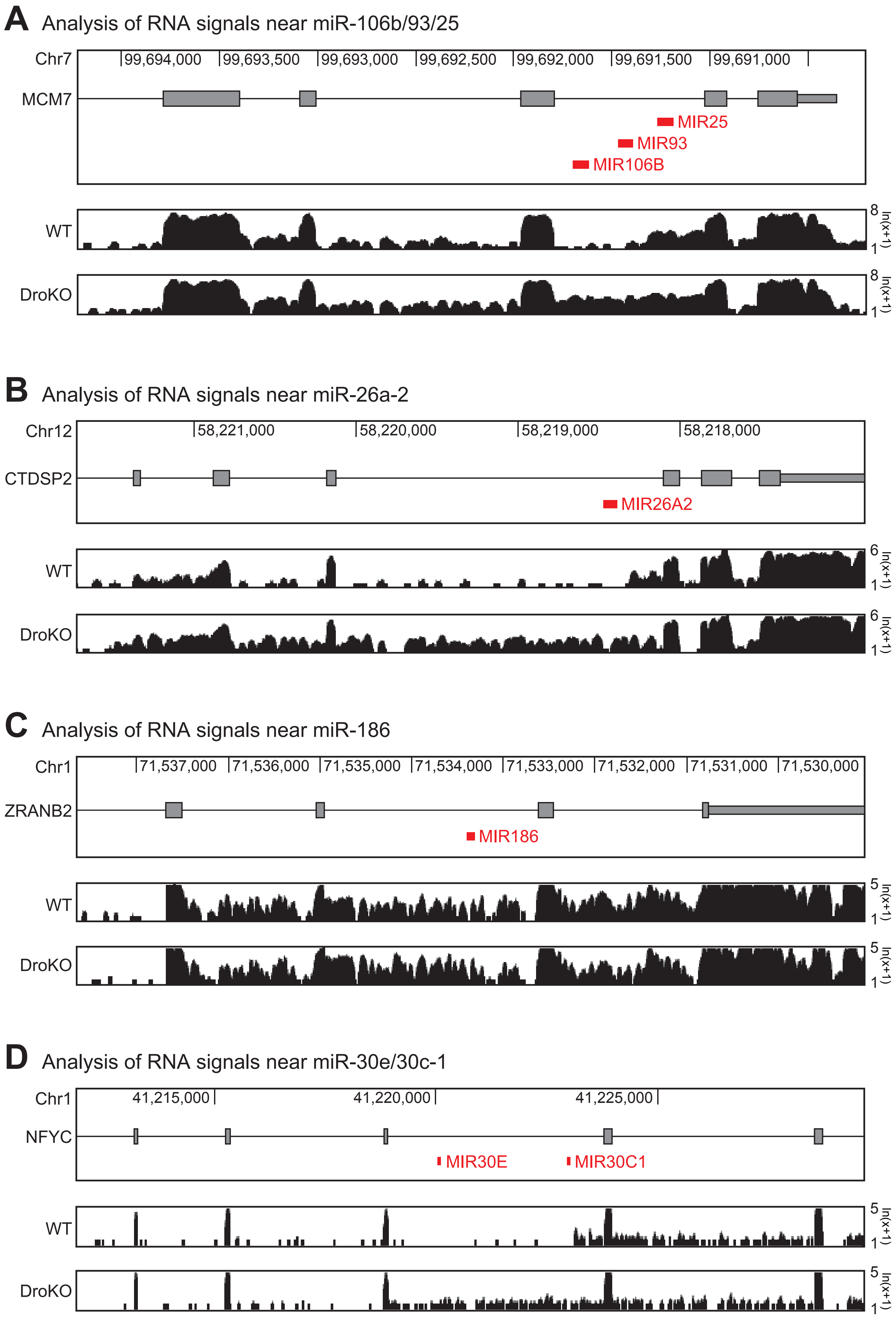
A
Analysis of RNA signals near miR-106b/93/25
Chr7
99,694,000
99,693,500
99,693,000
99,692,500
99,692,000
99,691,500
99,691,000
MCM7
MIR25
MIR93
MIR106B
WT
DroKO
8
1
ln(x+1)
B
Analysis of RNA signals near miR-26a-2
Chr12
58,221,000
58,220,000
58,219,000
58,218,000
CTDSP2
MIR26A2
WT
DroKO
6
1
ln(x+1)
C
Analysis of RNA signals near miR-186
Chr1
71,537,000
71,536,000
71,535,000
71,534,000
71,533,000
71,532,000
71,531,000
71,530,000
ZRANB2
MIR186
WT
DroKO
5
1
ln(x+1)
D
Analysis of RNA signals near miR-30e/30c-1
Chr1
41,215,000
41,220,000
41,225,000
NFYC
MIR30E
MIR30C1
WT
DroKO
5
1
ln(x+1)
