# Supplementary material for: Precise mapping of the transcription start sites of human microRNAs using DROSHA knockout cells
Source: BMC Genomics. 2016 Nov 11;17:908. doi: 10.1186/s12864-016-3252-7 (PMC5106785; doi:10.1186/s12864-016-3252-7)

**A** Size of intron/exon of miRNA host genes

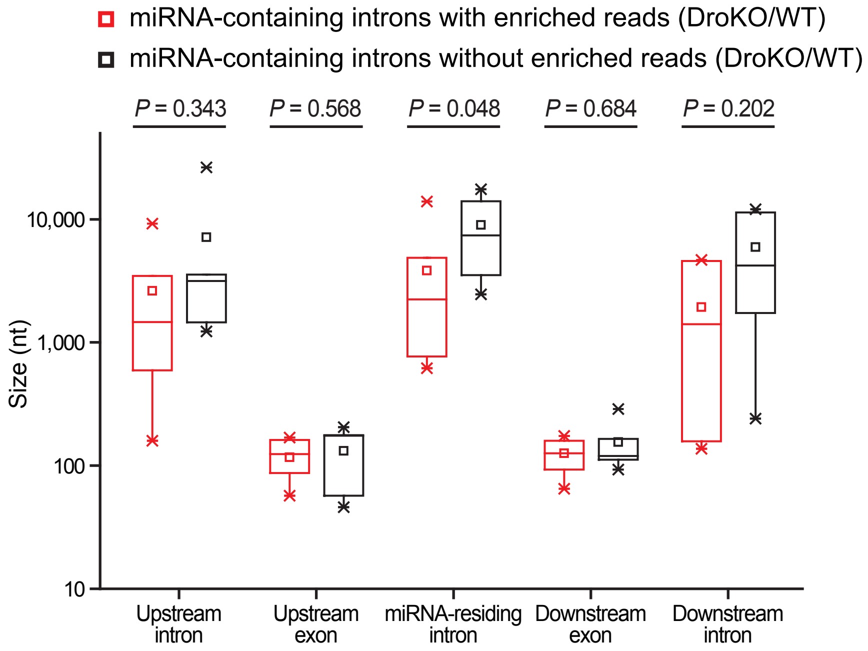


**B** Relative position of miRNA-containing introns within genes

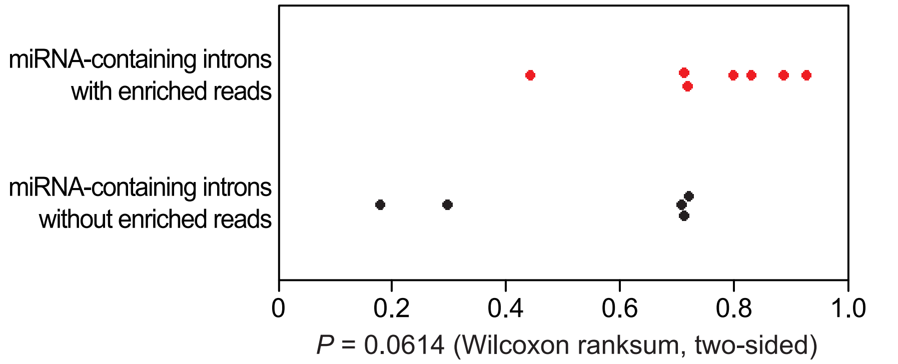

Supplement: Additional file 11: — The analyses of size and genomic location of miRNA-containing introns. (A) The size of each intron/exon was compared between the host genes with enriched reads at miRNA-containing introns and those without enriched reads. (B) The relative positions of miRNA-containing introns were also compared. P values were calculated by Wilcoxon rank-sum test. (PDF 121 kb) [file 12864_2016_3252_MOESM11_ESM.pdf]
